# Vector Autoregression for Forecasting the Number of COVID-19 Cases and Analyzing Behavioral Indicators in the Philippines: Ecologic Time-Trend Study

**DOI:** 10.2196/46357

**Published:** 2023-06-27

**Authors:** Angelica Anne Eligado Latorre, Keiko Nakamura, Kaoruko Seino, Takanori Hasegawa

**Affiliations:** 1 Department of Global Health Entrepreneurship Tokyo Medical and Dental University Tokyo Japan; 2 Department of Epidemiology and Biostatistics, College of Public Health University of the Philippines-Manila Manila Philippines; 3 Department of Integrated Analytics, Medical and Dental Data Science Center Tokyo Medical and Dental University Tokyo Japan

**Keywords:** COVID-19, forecasting, interest by the general public, mobility, surveillance, vector autoregression

## Abstract

**Background:**

Traditional surveillance systems rely on routine collection of data. The inherent delay in retrieval and analysis of data leads to reactionary rather than preventive measures. Forecasting and analysis of behavior-related data can supplement the information from traditional surveillance systems.

**Objective:**

We assessed the use of behavioral indicators, such as the general public’s interest in the risk of contracting SARS-CoV-2 and changes in their mobility, in building a vector autoregression model for forecasting and analysis of the relationships of these indicators with the number of COVID-19 cases in the National Capital Region.

**Methods:**

An etiologic, time-trend, ecologic study design was used to forecast the daily number of cases in 3 periods during the resurgence of COVID-19. We determined the lag length by combining knowledge on the epidemiology of SARS-CoV-2 and information criteria measures. We fitted 2 models to the training data set and computed their out-of-sample forecasts. Model 1 contains changes in mobility and number of cases with a dummy variable for the day of the week, while model 2 also includes the general public’s interest. The forecast accuracy of the models was compared using mean absolute percentage error. Granger causality test was performed to determine whether changes in mobility and public’s interest improved the prediction of cases. We tested the assumptions of the model through the Augmented Dickey-Fuller test, Lagrange multiplier test, and assessment of the moduli of eigenvalues.

**Results:**

A vector autoregression (8) model was fitted to the training data as the information criteria measures suggest the appropriateness of 8. Both models generated forecasts with similar trends to the actual number of cases during the forecast period of August 11-18 and September 15-22. However, the difference in the performance of the 2 models became substantial from January 28 to February 4, as the accuracy of model 2 remained within reasonable limits (mean absolute percentage error [MAPE]=21.4%) while model 1 became inaccurate (MAPE=74.2%). The results of the Granger causality test suggest that the relationship of public interest with number of cases changed over time. During the forecast period of August 11-18, only change in mobility (*P*=.002) improved the forecasting of cases, while public interest was also found to Granger-cause the number of cases during September 15-22 (*P*=.001) and January 28 to February 4 (*P*=.003).

**Conclusions:**

To the best of our knowledge, this is the first study that forecasted the number of COVID-19 cases and explored the relationship of behavioral indicators with the number of COVID-19 cases in the Philippines. The resemblance of the forecasts from model 2 with the actual data suggests its potential in providing information about future contingencies. Granger causality also implies the importance of examining changes in mobility and public interest for surveillance purposes.

## Introduction

The COVID-19 pandemic remains a significant threat to the health and well-being of people across the world. Stringent movement restrictions, including stay-at-home orders and closure of establishments, were one of the earliest interventions that were implemented to slow down viral transmission. Many countries also closed their borders, and up to this day, border measures such as quarantine and testing of travelers are still in place in some parts of the world [1]. Vaccines that are effective in reducing the severity of infection have been developed and have become a crucial element for governments in planning their respective lockdown exit strategies. However, more transmissible variants still emerge as the infection continues to spread despite these various nonpharmaceutical interventions, resulting in almost 700 million cases and over 6 million deaths globally as of September 2022 [2]. Thus, the occurrence of multiple outbreaks in many countries and areas is inevitable, and the questions on how to ease out restrictions while cutting the chain of transmission persist.

Surveillance is one of the key strategies for preventing and controlling the spread of communicable diseases such as COVID-19 [3,4]. The routine systematic collection and analysis of data inform the planning, implementation, and evaluation of public health programs and policies. It can serve as a system for generating early warning about imminent threats to public health or a monitoring system for tracking the attainment of program goals [5]. However, traditional surveillance systems are often retrospective due to delays in data gathering from disease reporting units. This leads to reactionary rather than preventive measures, especially during public health emergencies where information is scarce and the situation is rapidly changing [6].

Forecasting and multisource surveillance can fill the gap in information from traditional surveillance systems. Forecast, which is the quantitative predictions of health events or outcomes from previously reported data, can guide the timing and scale of disease prevention and control measures [6]. It aids the preparation and coordination of response by providing information that can be used for making critical decisions on allocating and deploying resources as well as developing preventive strategies [5]. On the other hand, unconventional sources of data complement traditional surveillance systems by rapidly capturing data about rare events and events that occur among populations that do not access the formal health care pathway. Mobility data, search trends, social media, and environmental data are additional sources of data that can help in early detection of unknown or emerging diseases [7]. In addition, these data sources allow the triangulation of evidence about an ongoing epidemic.

Time series modeling is one of the common methods for generating forecasts and has informed surveillance and response for communicable disease such as influenza and dengue [6,8,9]. Recent literature has also shown its potential use for forecasting COVID-19. Various univariate models have been used to predict the number of cases, deaths, and hospitalizations due to COVID-19 [10-14]. However, one of the criticisms of this approach is its inability to capture the interdependency of these parameters and hence, multivariate time series methods have been used to fill this gap [15,16]. Khan et al [15] jointly modeled the number of cases, hospitalizations, and deaths due to COVID-19 in Pakistan using vector autoregression (VAR). Rajab et al [17] used the same method in forecasting the pandemic in the United Arab Emirates, Saudi Arabia, and Kuwait, while Meimela et al [18] demonstrated the use of VAR in predicting together the number of cases and deaths in Indonesia.

Besides jointly forecasting potentially correlated variables, a primary application of VAR is investigating the relationship between variables [19,20]. Scholars have used VAR to describe the interaction of a variety of variables with the spread of SARS-CoV-2 using real-world data. A study conducted by Krishna [21] showed that population density improved the prediction of the number of COVID-19 cases in all major cities of India and suggested the possible variation in the environmental characteristics of each city as the relationship of temperature, air quality, and humidity with COVID-19 cases was found to be inconsistent. The interconnectedness of countries in terms of cross-border transmission was also exhibited in the works of Fitriani et al [22] and Milani [23]. Milani [23] further emphasized how international experiences affected the adaptation of response measures and gradual learning about SARS-CoV-2 as shocks in the number of cases in Italy or the United States brought about changes in risk perception and fear of unemployment in other countries. However, to the best of our knowledge, there is paucity of studies that explored the usefulness of behavioral indicators for forecasting and investigated the changes in the interrelationship of behavioral indicators with the trend of COVID-19 cases over time.

In this paper, we developed a VAR model using behavioral indicators such as change in mobility and public interest on the risk of contracting SARS-CoV-2. Our primary objective was to assess the use of the abovementioned behavioral indicators for forecasting the daily number of COVID-19 cases in the Philippines. The secondary objective was to investigate the relationship of the daily number of cases with change in mobility and public interest.

## Methods

### Study Design and Description of Variables

An etiologic, time-trend, ecologic study design was employed to forecast the daily number of reported COVID-19 cases in the National Capital Region (NCR). The NCR was selected as the study site because it is considered the epicenter of the pandemic in the Philippines, with a total of 1,175,949 cases, which comprise 31.8% of the recorded cases in the country [24]. It is also more homogenous than other regions in terms of urban-rural classification, as it is composed of 16 cities and only 1 municipality.

We computed the out-of-sample forecasts at multiple time periods during the resurgence of COVID-19. The first forecast period from August 11-18 coincides with the beginning of the resurgence of COVID-19 due to B.1617.2 (Delta) [24,25]. A total of 212 time points from January 11 to August 10, 2021, were used as the training data to obtain the out-of-sample forecast. We excluded the period of March-December 2020 in the analysis because the detection of variants of concerns, improvements in testing availability and strategy, and roll out of vaccines have only occurred in 2021.

After computing the out-of-sample forecast for August 11-18, we added data points to the training data set and performed recalibration to test the data toward the peak in the number of cases from September 15-22. Lastly, we used the data from June 1 to January 27 to forecast the number of cases during the spread of B.1.1.529 (Omicron) in NCR; the testing data is from January 28 to February 4.

Daily number of cases, change in mobility, and public interest on the risk of contracting COVID-19 were treated as endogenous variables since previous literature suggests the presence of a bidirectional relationship among these 3 variables [21,23]. These variables were also chosen to demonstrate the use of publicly available data for surveillance.

The data on the number of COVID-19 cases was collected from the COVID-19 case tracker of the Philippine Department of Health (DOH). The reported number of cases includes only those that were detected through reverse transcriptase–polymerase chain reaction (RT-PCR) in laboratories that were accredited by the DOH-Research Institute of Tropical Medicine [24].

We used the publicly available data from Google as an indicator of the behavior-related endogenous variables change in mobility and public interest on the risk of contracting SARS-CoV-2. Since 82.5% of the 10- to 64-year-old age group in the urban area use social media, big data source from Google has the potential to capture the changes of these variables in NCR over time [26]. Change in mobility was collected from Google’s Community Mobility Report. The Community Mobility Report is aggregated and anonymized GPS-derived data obtained from people who turned on the location reporting settings of their devices. Google categorized locations into 6 types, namely, workplaces, retail and recreation, transit stations, residential, parks, and groceries and pharmacies, based on the social distancing guidance. These time series data pertain to the percent change in mobility from the baseline value, which is the median value for the corresponding day of the week during the 5-week period from January 3 to February 6, 2020, for each type of place [27].

We retrieved the data on public interest on COVID-19 risk from Google Trends by using keywords related to the risk, symptoms, and prevention and control of COVID-19 such as “COVID-19 risk,” “COVID-19 control,” “COVID-19 variant,” “COVID-19 symptoms,” and “COVID-19 vaccine treatment” in a single query. Since the number of keywords is within the limit for viewing data in a single search, further iteration or transformation of the time series data was not necessary. The data from Google Trends are samples of the absolute search volume that were indexed on a scale ranging from 0 to 100 by dividing each point by the highest point in the time series. This means that the time points or dates with a value of 100 are interpreted as the date or period wherein the number of searches for the topic was highest [28]. In this study, the downloaded daily data from Google Trends were directly used in the analysis and were considered as a measure of the population’s interest on the risk of contracting COVID-19 in NCR over the study period.

### Analysis

After downloading the data for NCR from Google’s Community Mobility Report, we computed a combined mobility index for change in mobility [27]. We modified the method used by Wang et al [29], wherein the daily changes in mobility in the 6 categories of location were averaged, by excluding the data for residential places as well as groceries and pharmacies. Mobility in residential category was not included because its interpretation is different from the mobility measures in other types of location. Change in mobility in residential locations provides information on how the length of stay in residential places differed from baseline, while the data in other categories are interpreted as the change in volume of visitors from baseline [27]. On the other hand, mobility in grocery and pharmacies was excluded in the analysis because this category includes health care facilities and other places where basic needs are obtained, and people were not prohibited from entering these types of places under this category.

The daily number of COVID-19 cases was forecasted through VAR. VAR models *k* variables as a linear function of their own *p* lags and the *p* lags of the other *k*-1 variables. It is given by:



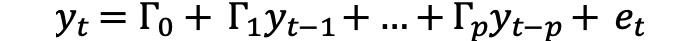



, where *y*_t_ is the (n×1) vector of endogenous variables, г_0_ is the (n×1) vector of intercept terms, г_p_ is the (n×n) vector of coefficients, and *e*_t_ is the (n×1) vector of white-noise disturbances with constant mean and variance.

Model estimation and forecasting using VAR involved five steps: (1) assessment of stationarity of variables; (2) lag length selection; (3) model estimation; (4) testing for autocorrelation, stability, and Granger causality; and (5) assessment of forecast accuracy [19,20]. All analyses were performed using STATA 14 (StataCorp) and the level of significance for all the tests was set at .05 [30].

Stationarity of the variables is a requirement for time series modeling. In this study, we determined the stationarity of each variable using the Augmented Dickey-Fuller (ADF) test. Rejection of the null hypothesis of the presence of a unit root signified that the data are stationary.

Selection of lag length is another critical step of VAR since the coefficients of each equation and the properties of the model depend on the lag length [19,20]. This was performed by combining knowledge on the epidemiology of SARS-CoV-2 with the results of information criteria measures. The lag with the smallest value for Akaike information criterion (AIC), Hannan-Quin information criterion (HQIC), and Schwarz Bayesian information criterion (SBIC) were considered as possible candidates for lag length of the VAR with a dummy variable for the day of the week. We selected the lag that captured the full cycle of data in relation to the incubation period of the virus for model estimation [19,20].

After selection of lag length for the VAR, we estimated each equation of the reduced VAR model using ordinary least squares. We tested the assumptions on stability and absence of residual autocorrelation of the estimated system of equations by checking the modulus of the resulting eigenvalues and through Lagrange multiplier test, respectively. Presence of a modulus of an eigenvalue of 1 or more signified that the VAR was unstable, while rejection of the null hypothesis of no residual correlation at a given lag length is interpreted as a violation of the assumption on residual autocorrelation.

Granger causality test was also performed to check whether inclusion of past values of changes in mobility and public interest improved the prediction of the number of cases. Rejection of the null hypothesis at the 0.05 level of significance suggested that at least one of the past values of the variable being tested was able to improve the prediction of the daily number of cases.

**Figure 1:**
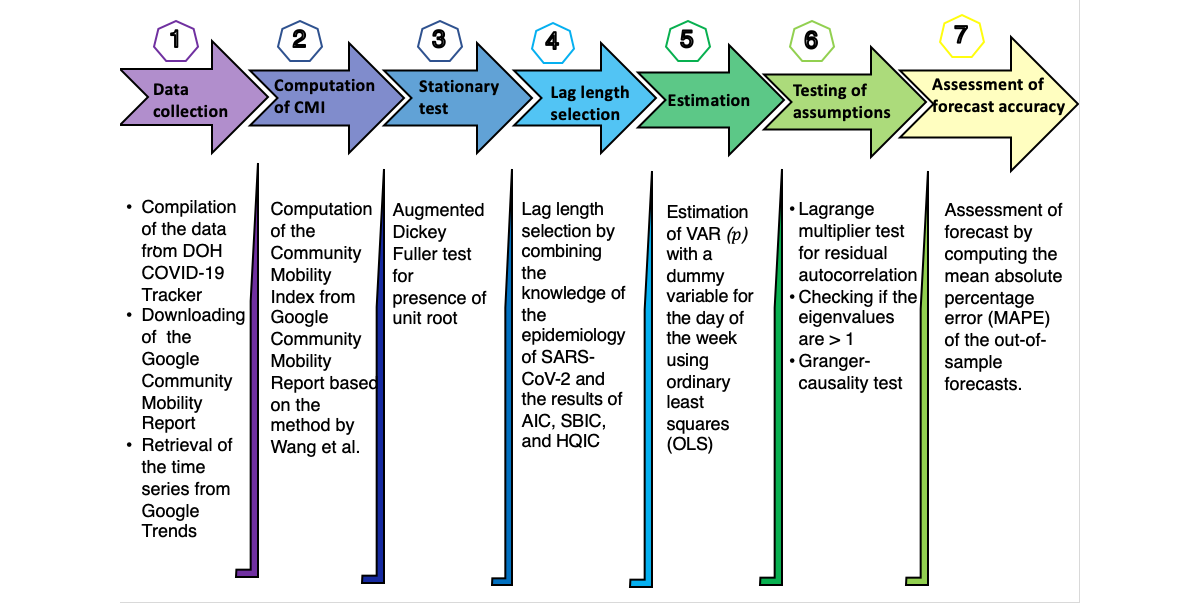
Steps in collecting and analyzing data. The combined mobility index (CMI) was determined based on Wang et al [29]. AIC: Akaike information criterion; DOH: Department of Health; HQIC: Hannan-Quin information criterion; MAPE: mean absolute percentage error; OLS: ordinary least squares; SBIC: Schwarz Bayesian information criterion; VAR: vector autoregression.

We obtained 2 VAR models for each forecast period using the steps described above. Model 1 contains change in mobility and number of cases as the endogenous variables and a dummy variable for the day of the week, while model 2 includes public interest as an additional endogenous variable. The forecast accuracy of these models was compared through the mean absolute percentage error (MAPE). A MAPE of less than 10% is interpreted as highly accurate forecast, while 11%-20%, 21%-50%, and more than 50% were considered good forecast, reasonable forecast, and inaccurate forecast, respectively [31]. Figure 1 summarizes the steps that were followed in building the VAR models.

### Ethical Considerations

The data acquired from Google Trends, Google Community Mobility Report, and DOH COVID-19 Tracker are secondary and contain no sensitive information. Thus, there was no need to secure an informed consent form from any individual. The research also used open-source data sets, and it is for this reason that the investigators did not apply for permission to access the databases. A proper citation was made to acknowledge the institutions that published the data for public use.

## Results

The results of the ADF test revealed that the individual time series do not contain a unit root, and therefore, integration or other methods to achieve stationarity are not necessary [19,20]. Table 1 shows the test statistic and the *P* values obtained from the test of stationarity.

After testing if the individual variable is stationary or not, we determined the lag length of each model. All measures of information criterion selected a lag length of 8 for model 1, with values of 20.37, 20.62 and 20.98 for AIC, HQIC, and SBIC, respectively. On the other hand, the second lag has the smallest SBIC value equal to 28.75, but the eighth lag was shown to have the lowest AIC and HQIC of 27.90 and 28.43, respectively, for model 2. Given the hypothesized seasonality of the 3 variables and the minimum incubation period of SARS-CoV-2, we favored the use of 8 lags for both model 1 and model 2. Hence, a VAR(8) was fitted to the daily data points (Table S1 in Multimedia Appendix 1).

We found that the model satisfied the assumptions for VAR after the postestimation hypothesis tests. The null hypothesis of the absence of residual autocorrelation was not rejected for all the lags that we tested. The result of the assessment of the stability of the VAR showed that the modulus of each eigenvalue is strictly less than 1 (Table S2 in Multimedia Appendix 2).

**Figure 2:**
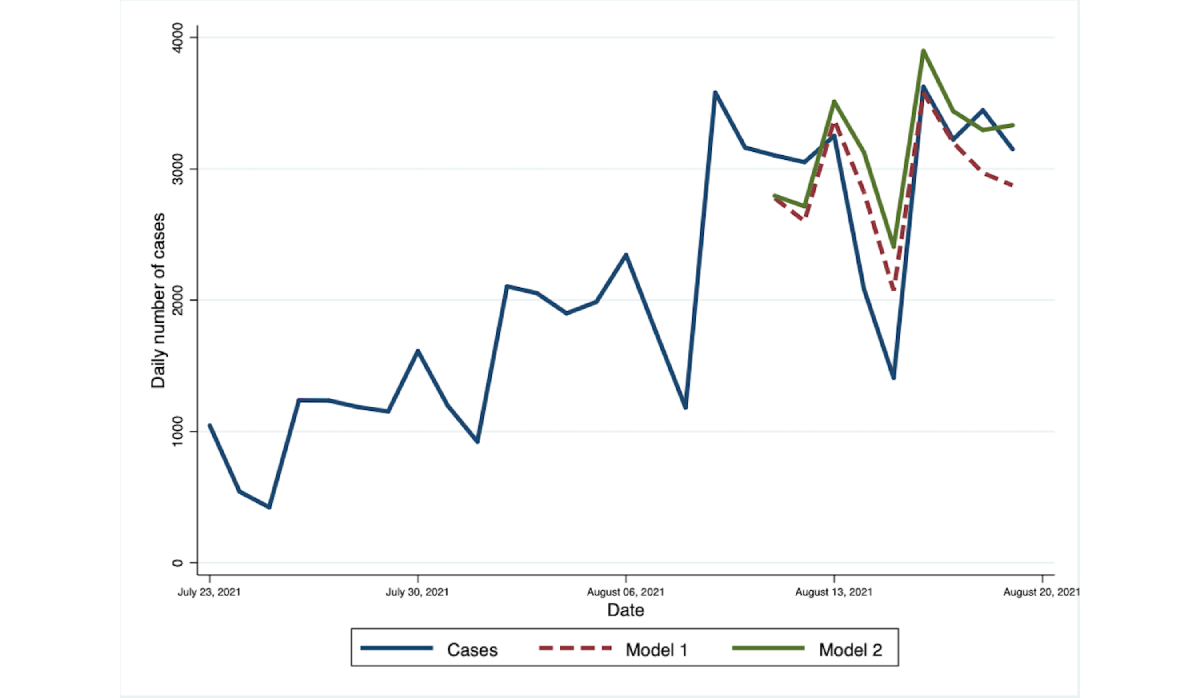
Out-of-sample forecasts from Model 1 and Model 2 and the observed daily number of cases in the National Capital Region, August 11-19, 2021. Model 1 contains change in mobility and number of cases as endogenous variables and a dummy variable for the day of the week. Model 2 includes all the variables in Model 1 with the addition of public interest as an endogenous variable. Observed Daily Number of Cases refers to the number of cases reported in the Philippine Department of Health’s COVID-19 Tracker.

**Figure 3:**
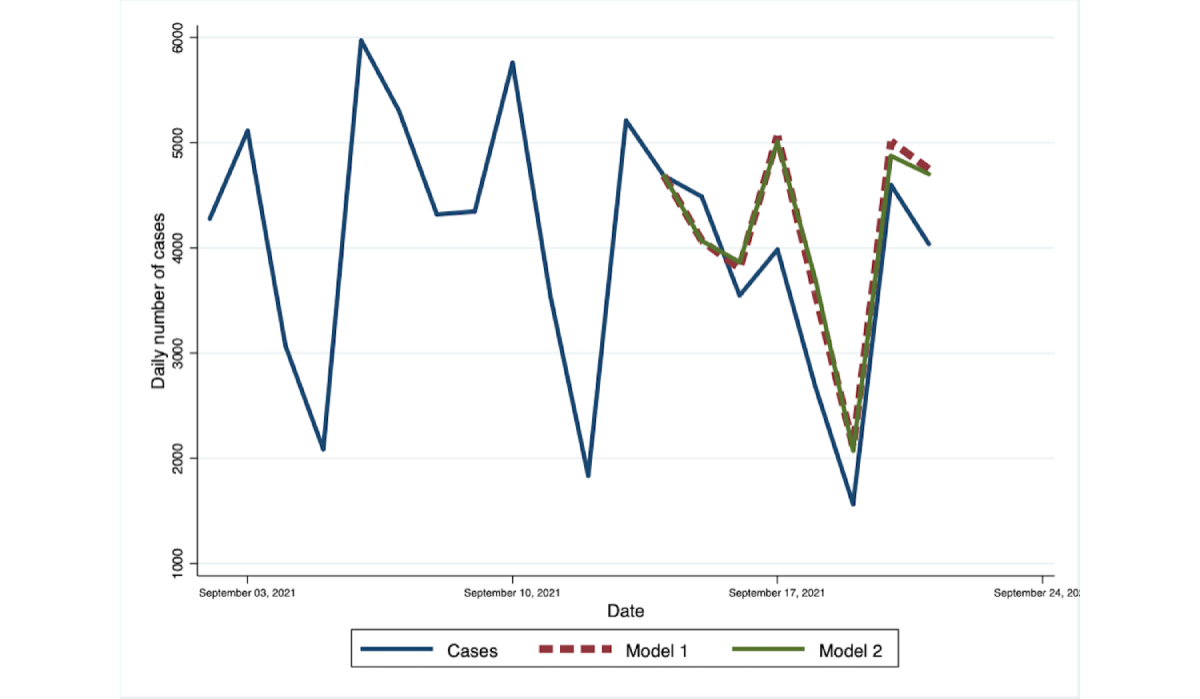
Out-of-sample forecasts from Model 1 and Model 2 and the observed daily number of cases in the National Capital Region, September 15-22, 2021. Model 1 contains change in mobility and number of cases as endogenous variables and a dummy variable for the day of the week. Model 2 includes all the variables in Model 1 with the addition of public interest as an endogenous variable. Observed Daily Number of Cases refers to the number of cases reported in the Philippine Department of Health’s COVID-19 Tracker.

**Figure 4:**
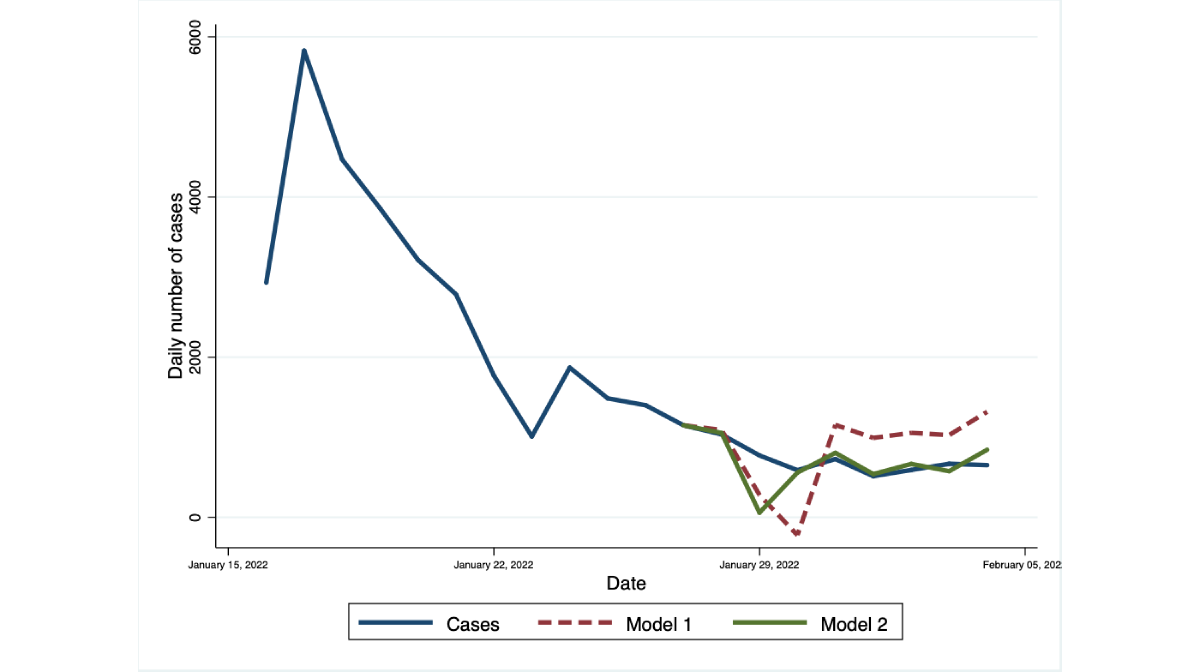
Out-of-sample forecasts from Model 1 and Model 2 and the observed daily number of cases in the National Capital Region, January 28 to February 4, 2022. Model 1 contains change in mobility and number of cases as endogenous variables and a dummy variable for the day of the week. Model 2 includes all the variables in Model 1 with the addition of public interest as an endogenous variable. Observed Daily Number of Cases refers to the number of cases reported in the Philippine Department of Health’s COVID-19 Tracker.

The VAR(8) for model 1 and model 2 was used to forecast the daily number of cases for 3 periods. Both models generated forecasts with similar trend and spike to the actual number of COVID-19 cases. However, model 1 obtained values that are closer to the reported number of cases during the first forecast period, with a MAPE of 15.9%. On the other hand, model 2 showed a slightly lower forecast error of 17.6% compared with 17.9% for model 1 for the forecast period of September 15-22. The forecasted peak in the number of cases occurred earlier compared with the actual data, despite the improvement in the overall forecast performance of model 2 during this period. The difference in the performance of the 2 models became substantial when we made the predictions for the number of cases from January 28 to February 4, as the accuracy of model 2 remained within reasonable limits (MAPE=21.4%), while model 1 became inaccurate (MAPE=74.2%). The out-of-sample forecast as compared to the actual number of cases during the 3 forecast periods are presented in Figures 2-4, while the summary of MAPE is in Table 2.

The result of Granger causality test for model 2 is shown in Table 3. It suggests that the relationship of the variables with the number of cases has changed over time. During the forecast period of August 11-18, only change in mobility was found to improve the forecasting of the daily number of cases. It also became more statistically significant for predicting the number of cases during the second and third forecast periods. On the other hand, public interest was also found to Granger-cause the daily number of cases during September 15-22, and this statistically significant result was also found for the VAR(8) that was used for forecasting the number of cases from January 28 to February 4.

**Table 1:** Results of Augmented Dickey-Fuller test.

Variable name	*z* statistic	*P* value
Public interest	–5.26	<.001
Change in mobility	–4.81	<.001
Number of cases	–3.23	.02

**Table 2:** Mean absolute percentage error of model 1 and model 2 for the forecast periods.

Forecast period	Mean absolute percentage error	
	Model 1, %	Model 2, %
August 11-18, 2021	15.9	21.1
September 15-22, 2021	17.9	17.6
January 28-February 4, 2022	74.2	21.4

**Table 3:** Results of Granger causality test for change in mobility and public interest across the forecast periods.

Forecast period	Public interest		Change in mobility	
	*F* statistic	*P* value	*F* statistic	*P* value
August 11-18, 2021	6.61	.60	24.27	.002
September 15-22, 2021	26.67	.001	28.62	<.001
January 28-February 4, 2022	23.36	.003	27.92	<.001

## Discussion

### Overview

The resulting model containing change in mobility and daily number of cases had a MAPE ranging from 15.9% to 74.2%, while the VAR(8) with public interest as an additional variable achieved MAPEs with values from 17.6% to 21.8%. This shows that the forecast accuracy of model 1 became inaccurate and weak over time, while model 2 remained within reasonable forecasting limits. The MAPE that was obtained in this study was not as low as the ones developed for other countries, and this may be partly explained by the use of behavioral indicators instead of number of deaths and number of hospitalizations which were used in other models. Despite this, the resemblance of the trend and the spike of cases based on the forecast with reported number of cases in the NCR of Philippines makes the model a potential tool for creating an early warning system. According to Allard [32], the value of forecasting does not lie in the ability of the model to predict the future with accuracy but in its potential to provide information on the contingencies that may potentially happen in the future. This makes the forecast results potentially useful, particularly for local government units and health managers who regularly update their response measures within a short to medium time frame during a health emergency.

The use of change in mobility and public interest on the risk of contracting COVID-19 enabled the investigation of how the population’s behavior and risk perception may affect the trend in the daily number of COVID-19 cases. This is one of the advantages of the model that we developed over previously published VAR models that focused only on forecasting using counts of cases and deaths, and environmental factors that are not easily modifiable. The Granger causality between public interest and daily number of cases, as well as change in mobility and daily number of cases, also support the recommendations on using nonconventional sources of data for surveillance. Google Trends reflects the topics that are being searched for or reviewed by internet users. It is for this reason that it has become a readily available way to track people’s attention and study the changes in their interest over time, especially in the field of advertisement and economics [23,28]. In epidemiology, public interest on COVID-19 can be a marker of ongoing but undetected infection over a specific period and location, or it can be a proxy measure for the population’s risk perception and intention to practice self-protection measures. Krishna [21] and Milani [23] both demonstrated the possibility of using internet search volume for words related to protective measures as signs of precautions taken by the population over a period of time. On the other hand, Walker and Sulyok [33], Lin et al [34], and Higgins et al [35] have shown the correlation between web-based traffic search trend and the number of reported cases and highlighted the potential of “infodemiology” for surveillance. However, these studies may have biased results, as correlation analysis did not account for the serial correlation of search trends and the number of cases. Previous studies have also focused on specific keywords, such as “handwashing,” “mask,” “loss of smell,” “fever,” etc, that are frequently used by the media, and thus, the results may have been driven by the media reports instead of the actual interest of the population on COVID-19. This study adds to the existing body of knowledge by providing evidence on the usefulness of search trends through a more statistically robust method and more general search terms related to the risk of acquiring COVID-19.

Although literature has documented the direct relationship between mobility and number of cases using simulation studies and analysis of aggregated mobility data [36-38], community quarantine alone was shown to be ineffective in curtailing the spread of infection and brought devastating economic and societal impact on different populations [39]. As the governments design their respective lockdown exit strategy, it is crucial to maintain the awareness of the population about the persistence of the threat due to the virus and sustain its interest in disease prevention and control measures [40]. The importance of providing attention to public interest was supported by the change in statistical significance of public interest for the prediction of the number of COVID-19 cases over time and the weaker performance of model 1 as public interest becomes important in predicting the number of cases for September 15-22, 2021, and January 28 to February 4, 2022. Friedman and Kuttner [41] recommended the assessment of the innovation accounting of variables once Granger causality hints a change in the dynamics of variable. Variance decomposition and impulse response analysis can aid in achieving better understanding of the interrelationships of the variables and, consequently, development of more appropriate policies. Hence, further investigation of how the number of cases will respond to changes in mobility and public interest may be warranted to improve the planning and implementation of prevention and response measures.

The variability of the MAPE during the 3 forecast periods can be attributed to several factors. The detection of local transmission of the Delta variant in all cities and municipalities of NCR had just been reported a week before the time that coincided with first forecast period while the second forecast period was the peak of the resurgence [25]. Given the change in the dominantly circulating variant, with the Delta variant being markedly more transmissible than previous ones, it was expected that the epidemic patterns of COVID-19 during August 11-18 and September 15-22 would also be different [42]. It is possible that the changes in the epidemic pattern of COVID-19 were better captured by the training data that were used for forecasting the cases from September 15-22 (Multimedia Appendices 3-5). The increase in statistical significance of public interest in predicting the number of cases was also observed during the second forecast period. These 2 factors may have contributed to the improvement of the forecast performance of model 2 during the second forecast period. On the other hand, the faster transmission rate of the Omicron variant during the third forecast period led to a sharper rise and fall in the number of COVID-19 cases, and this may be expected to increase the MAPE for January 28 to February 4, 2022. It is worth noting that despite the change in trend of the number of cases, the forecast from model 2 generated values that are very close to the actual number of cases except for the point for January 29. This single value led to a bigger absolute difference that skewed the average of the absolute percentage error.

To the best of our knowledge, this is the first study that attempted to forecast the number of COVID-19 cases in the Philippines using time series analysis. Forecasting is not part of routine surveillance in the Philippines. This may be partly due to challenges in data management and analysis such as limited number of skilled data analysts, complicated data compilation, and lack of clear guidelines on analysis that low-middle-income countries are experiencing [5,43]. The modeling approach in this study was kept simple to make it feasible to transfer the knowledge and skills of data analysis to end users. It is for this reason that a simple method on computing the combined mobility index was preferred over more advanced techniques for creating indices such as Principal Component Analysis. In addition, geographical variability is an important consideration in determining the scope of time series analysis [44] and hence, the forecasting approach was applied to the NCR instead of the whole Philippines.

Despite the capacity of the resulting model to forecast the number of cases, this research has some limitations. The data source included those cases that were confirmed to be positive through RT-PCR by DOH-accredited laboratories, and as a consequence, underreporting of cases is possible given that the current case definition for COVID-19 includes cases that were detected through antigen tests. Second, since the data sources for public interest and change in mobility are affected by internet usage, there is a possibility that the data may not be representative of behavior-related indicators for the segment of the population that does not frequently use the internet. Despite this, the data sources remain useful in investigating risk perception and behavior at the aggregate level. Third, it was assumed that public interest, change in mobility, and daily number of cases were fixed variables throughout the period of coverage. It may be possible for these indicators to vary over time, and models that allow time-varying variables can be explored in future research. Nevertheless, the assumption on uniform mean and variance is justified given the results of the ADF test that suggested the stationarity of these variables. Finally, the demonstration of the modeling approach was done using publicly available data to ensure the ease of data collection and model recalibration, which are important considerations in real-life applications during health emergencies. Due to this, other variables without readily available data at the time when the study was conducted were not included in the study. This may also help explain why the MAPE of the models were not as low as the models in other countries.

To improve the forecast performance, it is recommended for future researchers to extend the model by incorporating additional endogenous variables, such as vaccine coverage and testing rate, that may be postulated to have a bidirectional relationship with the variables in this study. Weather-related or meteorological variables may also be added to the model, as these variables may affect public interest, change in mobility, and number of cases.

### Conclusions

To the best of our knowledge, this is the first study that forecasted the number of COVID-19 cases and explored the relationship of behavioral indicators with the number of COVID-19 cases in the Philippines. The resemblance of the forecasts from model 2 with the actual data suggests its potential in providing information about future contingencies. The results of Granger causality test also exhibit the importance of public interest in forecasting the number of cases and signify the importance of paying attention to the level of public interest in addition to changes in mobility. Hence, the findings of this study are indicative of the potential of forecasting and analysis of nonconventional data sources to complement the traditional surveillance system.

## Appendix

Multimedia Appendix 1 Supplementary Table 1. Results of Lag Length Selection for Models 1 and 2.

Multimedia Appendix 2 Supplementary Table 2. Results of Stability Test for Models 1 and 2.

Multimedia Appendix 3 Supplementary Figure 1. Time series data of the observed daily number of cases in the National Capital Region, Philippines from 1 June to 30 Sept 2021. Observed daily number of cases pertains to the number of reported cases in the Philippine-DOH COVID-19 Tracker.

Multimedia Appendix 4 Supplementary Figure 2. Time series data of public interest on the risk of contracting SARS-CoV-2 in the National Capital Region, Philippines from 1 June to 30 Sept 2021. Public interest refers to the data generated by Google trend after a single query containing keywords related to risk as well as prevention and control of COVID-19.

Multimedia Appendix 5 Supplementary Figure 3. Time series data of change in mobility in the National Capital Region, Philippines from 1 June to 30 Sept 2021. Change in mobility is the average of the changes in mobility measures in four different types of location.

## Abbreviations

ADF: Augmented Dickey-Fuller
AIC: Akaike information criterion
DOH: Department of Health
HQIC: Hannan-Quin information criterion
MAPE: mean absolute percentage error
NCR: National Capital Region
RT-PCR: reverse transcriptase–polymerase chain reaction
SBIC: Schwarz Bayesian information criterion
VAR: vector autoregression

## Data Availability

The data sets generated and analyzed during this study are available from the corresponding author on reasonable request.
