# Supplementary material for: Vector Autoregression for Forecasting the Number of COVID-19 Cases and Analyzing Behavioral Indicators in the Philippines: Ecologic Time-Trend Study
Source: JMIR Form Res. 2023 Jun 27;7:e46357. doi: 10.2196/46357 (PMC10337462; doi:10.2196/46357)
Supplement: Multimedia Appendix 1 [file formative_v7i1e46357_app1.docx]

Table 1. Results of Lag Length Selection for Models 1 and 2.

| Lag | Model 1 | | | Model 2 | | |
| --- | --- | --- | --- | --- | --- | --- |
|  | AIC | HQIC | SBIC | AIC | HQIC | SBIC |
| 0 | 23.5089 | 23.5364 | 23.5768 | 31.0475 | 31.0887 | 31.1493 |
| 1 | 21.0746 | 21.1296 | 21.2103 | 28.4978 | 28.6009 | 28.7523 |
| 2 | 20.9446 | 21.0271 | 21.1482 | 28.3425 | 28.5074 | **28.7496** |
| 3 | 20.8631 | 20.9730 | 21.1345 | 28.2974 | 28.5241 | 28.8573 |
| 4 | 20.8170 | 20.9544 | 21.1563 | 28.2715 | 28.5601 | 28.984 |
| 5 | 20.8018 | 20.9667 | 21.2090 | 28.2776 | 28.628 | 29.1428 |
| 6 | 20.6927 | 20.8851 | 21.1678 | 28.1545 | 28.5668 | 29.1725 |
| 7 | 20.5961 | 20.8160 | 21.1390 | 28.1058 | 28.5799 | 29.2765 |
| 8 | **20.3737** | **20.6211** | **20.9845** | **27.8971** | **28.4331** | 29.2205 |
| 9 | 20.4027 | 20.6775 | 21.0813 | 27.946 | 28.5438 | 29.4221 |
| 10 | 20.4250 | 20.7273 | 21.1715 | 27.9651 | 28.6248 | 29.5939 |
| 11 | 20.4566 | 20.7864 | 21.2710 | 28.0384 | 28.7599 | 29.8199 |
| 12 | 20.4611 | 20.8184 | 21.3434 | 28.0755 | 28.8588 | 30.0096 |
| 13 | 20.4842 | 20.8690 | 21.4343 | 28.127 | 28.9722 | 30.2138 |
| 14 | 20.5133 | 20.9256 | 21.5313 | 28.1629 | 29.0699 | 30.4024 |
| 15 | 20.4977 | 20.9374 | 21.5835 | 28.1847 | 29.1535 | 30.5769 |
| 16 | 20.4665 | 20.9338 | 21.6202 | 28.1682 | 29.1989 | 30.7131 |
| 17 | 20.4636 | 20.9583 | 21.6851 | 28.1802 | 29.2727 | 30.8778 |
| 18 | 20.4669 | 20.9891 | 21.7563 | 28.1931 | 29.3475 | 31.0434 |
| 19 | 20.4442 | 20.9939 | 21.8015 | 28.2074 | 29.4236 | 31.2104 |
| 20 | 20.4373 | 21.0145 | 21.8624 | 28.1604 | 29.4385 | 31.3161 |
