# Supplementary material for: Vector Autoregression for Forecasting the Number of COVID-19 Cases and Analyzing Behavioral Indicators in the Philippines: Ecologic Time-Trend Study
Source: JMIR Form Res. 2023 Jun 27;7:e46357. doi: 10.2196/46357 (PMC10337462; doi:10.2196/46357)
Supplement: Multimedia Appendix 2 [file formative_v7i1e46357_app2.docx]

Table 2. Results of Stability Test for Models 1 and 2.

| Model1 | | | | Model 2 | | | |
| --- | --- | --- | --- | --- | --- | --- | --- |
| Eigenvalue | | | Modulus | Eigenvalue | | | Modulus |
| 0.954058 |  |  | 0.954058 | 0.9575006 |  |  | 0.957501 |
| -0.2106956 | + | .8943314i | 0.918815 | -0.209998 | + | .8937761i | 0.918115 |
| -0.2106956 | - | .8943314i | 0.918815 | -0.209998 | - | .8937761i | 0.918115 |
| -0.8338958 | + | .3731909i | 0.913594 | -0.8347346 | + | .3784691i | 0.916526 |
| -0.8338958 | - | .3731909i | 0.913594 | -0.8347346 | - | .3784691i | 0.916526 |
| 0.5223867 | + | .7363196i | 0.902804 | 0.9037246 |  |  | 0.903725 |
| 0.5223867 | - | .7363196i | 0.902804 | 0.5128382 | + | .723726i | 0.887008 |
| 0.8990446 |  |  | 0.899045 | 0.5128382 | - | .723726i | 0.887008 |
| 0.4116247 | + | .6839764i | 0.798285 | 0.4400154 | + | .7241047i | 0.847314 |
| 0.4116247 | - | .6839764i | 0.798285 | 0.4400154 | - | .7241047i | 0.847314 |
| -0.661539 | + | .3476735i | 0.747336 | -0.8339202 |  |  | 0.83392 |
| -0.661539 | - | .3476735i | 0.747336 | 0.8282683 |  |  | 0.828268 |
| -0.1404736 | + | .7297602i | 0.743157 | -0.2068291 | + | .7820896i | 0.808976 |
| -0.1404736 | - | .7297602i | 0.743157 | -0.2068291 | - | .7820896i | 0.808976 |
| 0.7288534 | + | .1143471i | 0.737769 | 0.6001795 | + | .5218484i | 0.795325 |
| 0.7288534 | - | .1143471i | 0.737769 | 0.6001795 | - | .5218484i | 0.795325 |
| 0.954058 |  |  | 0.954058 | 0.7785171 | + | .03154436i | 0.779156 |
| -0.2106956 | + | .8943314i | 0.918815 | 0.7785171 | - | .03154436i | 0.779156 |
| -0.2106956 | - | .8943314i | 0.918815 | -0.5287523 | + | .5457652i | 0.759894 |
| -0.8338958 | + | .3731909i | 0.913594 | -0.5287523 | - | .5457652i | 0.759894 |
| -0.8338958 | - | .3731909i | 0.913594 | 0.06108591 | + | .7560815i | 0.758545 |
| 0.5223867 | + | .7363196i | 0.902804 | 0.06108591 | - | .7560815i | 0.758545 |
| 0.5223867 | - | .7363196i | 0.902804 | -0.659754 | + | .3119315i | 0.729778 |
| 0.8990446 |  |  | 0.899045 | -0.659754 | - | .3119315i | 0.729778 |
