# Supplementary figures and images for: Vector Autoregression for Forecasting the Number of COVID-19 Cases and Analyzing Behavioral Indicators in the Philippines: Ecologic Time-Trend Study
Source: JMIR Form Res. 2023 Jun 27;7:e46357. doi: 10.2196/46357 (PMC10337462; doi:10.2196/46357)

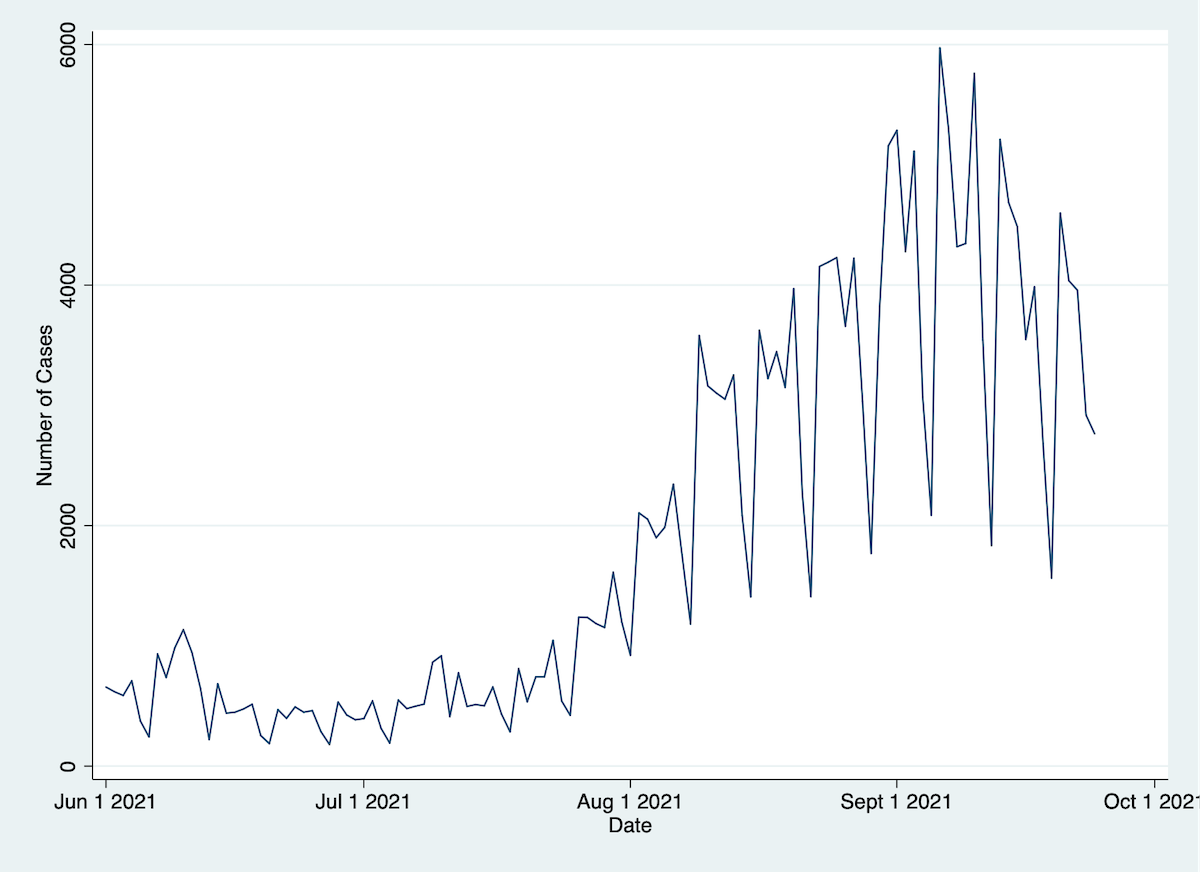

Supplement: Multimedia Appendix 3 [file formative_v7i1e46357_app3.png]

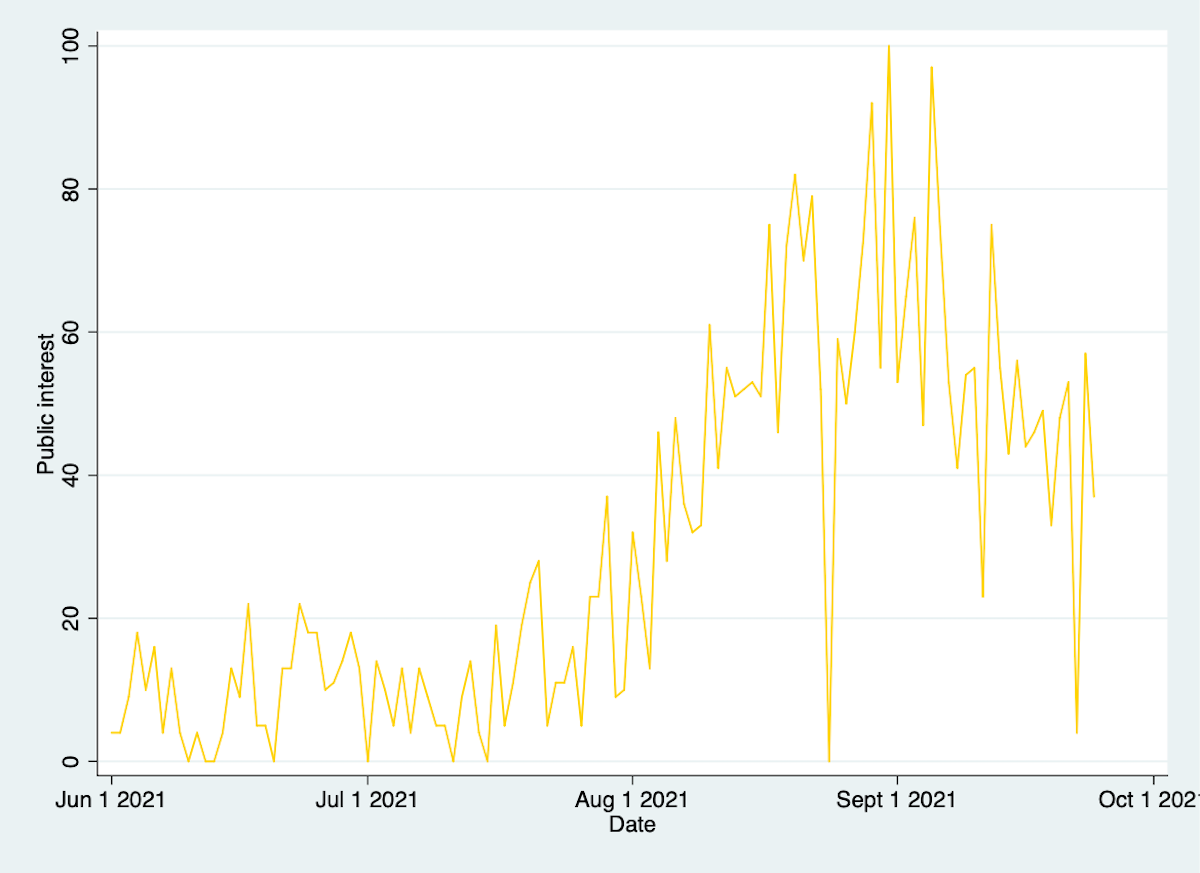

Supplement: Multimedia Appendix 4 [file formative_v7i1e46357_app4.png]

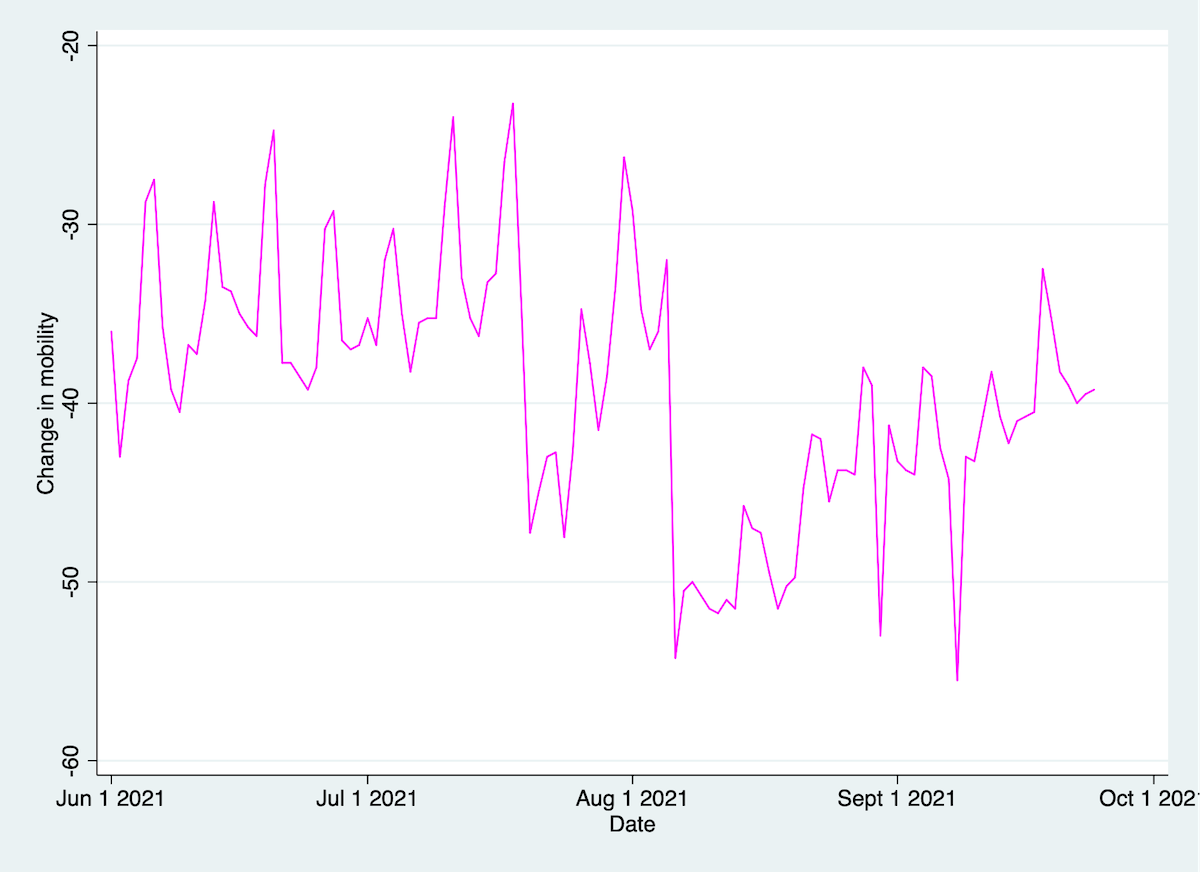

Supplement: Multimedia Appendix 5 [file formative_v7i1e46357_app5.png]
